# On the Arsenical Mineral Water of Whitbeck, Cumberland

**Published:** 1860-10

**Authors:** Arthur H. Church


### ARTICLE XXI.

### *On the Arsenical Mineral Water of Whitbeck, Cumberland.*

By Arthur H. Church, F. C. S.

The recent reliable accounts of arsenic eating in Styria, the controversy as to the effect on the Thames water of arsenical perchloride of iron, and the detection of arsenic in numerous mineral waters and deposits invite special notice to the occurrence of this element. And now that the influence on the animal economy of arsenic in minute but repeated doses is attracting so much attention, I think that the following account, incomplete as it at present is, of an arsenical drinking water will prove interesting. My attention was first directed to the subject by the Rev. Mr. Wilkin of Booth, to whom, as well as to the Rev. Mr. Ormandy of Whitbeck and Dr. Fidler of Whitehaven, I am indebted for much information and assistance.

From the northern and western sides of Black Combe, a mountain in the southern part of Cumberland, situated near the sea, numerous streams or becks originate : I believe that only one of these exhibits any marked peculiarity. Whitbeck, such is the name of this stream, is fed by several small springs, and it was from the source of the most southerly of these where it rises from the ground, and at an elevation of about 900 feet from the sea, that I obtained a specimen of the water for examination.

On the 29th of June in the present year, the water, at the ti'ne of collection, had a temperature of 8° 5′ C. the air being 10° 6′. The reaction of the water as it issues from the earth was faintly but unmistakably alkaline : on testing the water after ebullition the effect was more decided. The water from many other sources in the neighborhood of Whitbeck, where decomposing granite is of common occurrence, has an alkaline reaction. A large and deep pool in the course of Whitbeck towards the sea shows the color of the water to be a rich clear greenish blue. The water, on examination, gave distinct indications of the presence of arsenic. This element, which here probably exists as an alkaline arsenite, occurs not as a mere trace, but in determinable quantity. I have not yet ascertained the amount present, but hope to do so shortly, when I have obtained specimens of the water collected at different seasons of the year. I have satisfied myself, however, that in some seasons of the year the quantity present approaches a good fraction of a grain of arsenic (metallic) in each gallon of water. At the same time I am desirous of furnishing complete analyses of some interesting minerals obtained from the vicinity of the spring. For on ascending the gulley, a few yards above the source of Whitbeck, we arrive at the entrance to a mine, which, some years ago, was worked for cobalt and copper, and is now again being searched. Here I obtained very rich and massive silver-white arsenical cobalt ore, and also copper pyrites. The neighborhood for some miles is in fact rich in minerals. Dr. Fidler writes: "Almost immediately behind Whitehaven Parsonage a sulphur vein crops out, a continuation of the same vein that is being worked at Under Hill, but whether it exists in any quantity I do not know. There are three or four copper veins in a ravine behind Whitehaven Mill, one of which has been tried some twelve or fifteen fathoms below the surface." Baryta, also, has been found, I am told, above the source of Whitbeck in the mine above mentioned. It will be seen that the arsenic in the water of Whitbeck is thus most probably derived from the veins of arsenical cobalt ore through which it percolates.

The arsenical water is *habitually used for every purpose* by the inhabitants of the little village of Whitbeck, and, as far as I can learn, with beneficial rather than injurious results. But it is remarkable that Whitbeck, though in every respect suitable for trout, is the only stream in the neighborhood from which that fish is absent; eels, however, have been found in it. Ducks will not live if confined to this arsenical water. When the railway was being carried past Whitbeck, the first use of the water quickly produced the usual marked effect on the throats both of the men and horses employed on the works. The soreness of mouth from which they at first suffered soon, however, disappeared, and in the horses gave place to that sleekness of coat assigned as one of the effects produced by the administration of arsenic. It is a question how far the rosy looks of the Whitbeck children and the old age which a large proportion of the inhabitants of the village attains are to be attributed to the arsenic present in the water they drink.—*Chemical News.*

